# Fecal Incontinence after Severe Brain Injury: A Barrier to Discharge after Inpatient Rehabilitation?

**DOI:** 10.3390/neurolint15040084

**Published:** 2023-10-31

**Authors:** Laura Pelizzari, Elena Antoniono, Donatella Giraudo, Gianluca Ciardi, Gianfranco Lamberti

**Affiliations:** 1 Department of Rehabilitative Medicine, AUSL Piacenza, 29017 Fiorenzuola d’Arda, PC, Italy; l.pelizzari@ausl.pc.it (L.P.); gianluca.ciardi@unipr.it (G.C.); 2 Neurorehabilitation Unit, AUSL CN1, 12045 Fossano, CN, Italy; elena.antoniono@aslcn1.it; 3 Department of Urology, IRCCS San Raffaele Scientific Institute, 20127 Milano, MI, Italy; giraudo.donatella@hsr.it

**Keywords:** fecal incontinence, brain injuries, rehabilitation outcome

## Abstract

Background: In this study, we aimed to investigate the incidence of fecal incontinence (FI) after severe acquired brain injuries (sABIs) and to determine whether this symptom can lead to an inability to return home after rehabilitation. Methods: This was a retrospective observational cohort study. In total, 521 acute sABI inpatients were enrolled from the Department of Neurorehabilitation at an academic tertiary care hospital. Patients were divided into two groups, with and without FI, at the end of the rehabilitation phase. The primary and secondary endpoints were the incidence of persistent FI and any difference in the discharge destination. Results: Upon admission, new-onset FI was found in 443 (85%) patients, of which 38% had traumatic sABI. Moreover, 62.7% of all patients had FI upon admission. At discharge, 53.3% (264/495) of patients still had FI. Of these, 75.4% (199/264) had a Rancho Level of Cognitive Functioning Scale (LCFS) ≥3. A statistically significant correlation between FI at discharge and the presence of frontal lesions, autonomic crises, and increased LCFS scores was noted. Among the patients discharged to their homes, the proportion with persistent FI was lower (34% vs. 53.3). Conclusions: FI was significantly persistent after sABI, even after recovery from unconsciousness, and must be considered as a consequence of, rather than an independent risk factor for, unfavorable outcomes.

## 1. Introduction

A severe acquired brain injury (sABI) is usually defined as a traumatic or non-traumatic pathologic condition of the central nervous system (CNS) [1]. Following sABI, some patients can suffer from a state of coma (Glasgow Coma Scale ≤8) of variable duration, with a broad spectrum of impairments affecting physical and neurocognitive functioning, representing a significant cause of lifelong disability [2]. One of the most disabling and self-limiting complications is represented by altered nervous control of the bowel. This is known as neurogenic bowel dysfunction (NBD), which refers to the impairment of voluntary control over bowel function caused by CNS disorders. This condition manifests as a range of bowel symptoms, primarily characterized by fecal incontinence (FI) and/or constipation [3,4].

### 1.1. Neurologic Bowel System Control

The CNS plays a pivotal role in regulating various aspects of gastrointestinal regulation, including muscular processes, sensory perception, storage, and excretion [5]. The CNS and the enteric nervous system (ENS) engage in an intricate and ongoing connection. This interaction mostly occurs through the sympathetic prevertebral ganglia, pelvic, and vagus nerve pathways, which are located within the gastroenteric wall. The CNS plays a crucial role in regulating contractile and secretive functions in the upper gastrointestinal tract. Additionally, it is engaged in controlling motility in the lower tract, blood flow, and electrolyte transfer through reflex circuits mediated by ENS neurons. The ENS is the primary determinant in regulating intestinal function. It is comprised of around 500,000 neurons that are distributed throughout Meissner’s plexus, which governs intestinal secretions, and Auerbach’s plexus, which controls the motor activity of the entire intestine [6].

The intricate neural system possesses the ability to integrate various reflex activities throughout the digestive tract, exhibiting both excitatory and inhibitory functions. This characteristic highlights its independence from both the central nervous system and the peripheral nervous system. This particular circumstance enables us to accurately designate it as the “enteric nervous system” [7].

As can be inferred from the control mechanisms of gastrointestinal function, conditions related to CNS (central nervous system) alterations are often accompanied by intestinal neuronal dysfunctions.

The CNS governs defecation by exerting control through lumbosacral centers in the spinal cord [7]. The regulation of the anorectal system’s physiological function is facilitated by brain control in conjunction with anatomical structures and the innervation of both somatic and visceral peripheral components. In contrast to the extensive literature on spinal and peripheral innervation, our understanding of brain mechanisms governing anorectal continence remains limited [8]. The rectum functions as a storage compartment for both solid and liquid fecal matter, as well as the gases generated by the small and large intestines. Its primary role is to facilitate the effective evacuation of these contents. The presence of both smooth and striated muscle sphincters contributes to fecal continence maintenance. The cerebral structures responsible for maintaining urine continence also play a role in regulating fecal continence and evacuation mechanisms [9].

The physiological process of filling and emptying, which is subject to voluntary control, is contingent upon the transmission of information from the periphery to the brain. Any circumstance that interferes with the cognition, transmission, or processing of these data at the brain level has the potential to result in malfunction of the lower gastrointestinal tract [10,11,12].

The progress made in imaging techniques has facilitated the acquisition of knowledge pertaining to the cerebral regions that govern anorectal continence. The condition of rectal distension, which is similar to the introduction of a fecal bolus due to a high-amplitude propagated contraction (HAPC), elicits bilateral activity in the insula, anterior cingulate gyrus, secondary somatosensory cortex, and thalamus [13,14]. Motor areas such as M1, the supplementary motor area, and the cerebellum are specifically activated alone in response to anal stimulation. This activation is most likely a reflexive reaction to the expansion of the rectum, and it typically happens with a delay of around six seconds. Passive fecal continence is established through reflex motor activity, which serves to confine the fecal bolus within the rectal ampulla. The activation of the motor cortex in the supplementary motor region, as well as the primary somatosensory cortex and insula, occurs when the external anal sphincter undergoes voluntary contraction, particularly when this contraction is repeated [15].

Recent research has additionally demonstrated the simultaneous activation of brain regions responsible for regulating the external anal sphincter and the regulatory regions associated with the long flexor of the hallux [7]. The intricate coordination of cerebral functions, including continence, lower limb movement, and respiration, highlights the intricate nature of the control systems responsible for maintaining continence at the brain level. This integration appears to be linked to the physiological imperative of sustaining continence [16].

The confirmation of the overlap control between intestinal and bladder functions is supported by the control pathways present in the brainstem and spinal cord, as well as the peripheral innervation facilitated by the pudendal nerve, which is shared by both functions [17].

There is evidence that suggests the existence of a pontine defecation center, similar to the pontine micturition center (PMC), which regulates the distal colon, rectum, and internal anal sphincter. Additionally, the pontine continence center (PCC) seems to be responsible for controlling the external anal sphincter to maintain fecal continence [18].

### 1.2. Rationale and Aim of This Work

To date, limited information is available about FI persistence and risk factors in patients after sABI. Authors have also described that, during the acute phase, FI occurs in 23–70% of the sABI population [3,4,5,6] and persists in the chronic phase in 5–15% of cases [7,8,9,10,11,12,13,14,15,16]. Constipation has been reported in the chronic phase in 23% of patients [17]. Impaired consciousness can make bowel continence assessment difficult, and in critically ill patients, enteral nutrition (EN) [18] is associated with diarrhea, which is one of the most frequent causes of FI [19,20], often as a side effect of other treatments (e.g., antibiotics, osmolar compounds, cardiac drugs) in intensive care units [21]. Incontinence can prolong a patient’s stay in a rehabilitation setting and substantially hinder recovery from acquired brain injury [22,23,24,25]. Furthermore, FI can also lead to adverse effects such as skin disintegration, decubitus ulcers, and skin infections [23].

Thus, in the present study, we aimed to better understand FI incidence and its effects on rehabilitation outcomes after acute brain injury rehabilitation, to identify clinical variables associated with it, and to determine whether FI is an independent risk factor for poor functional recovery and for the discharge setting.

## 2. Materials and Methods

This was a retrospective and observational cohort study conducted in accordance with the STROBE guidelines statement [26]; it was conducted in a tertiary care rehabilitation hospital. According to the Aven guidelines, no ethics committee permission was required since the collection and subsequent processing of data was carried out anonymously, agglomerated, and not traceable to individual patients [27]. Despite the retrospective nature of this study, informed consent was collected from each patient/legal guardian at the beginning of the research process; this procedure ensured patients’ agreement to use their data in a clinical scope (inpatient rehabilitation procedures) as well as a research scope [27].

Data were collected from a medical chart review of all consecutive acute traumatic and non-traumatic sABIs from January 2005 to December 2018, regardless of the severity of the cognitive impairment. Patients with a history of other neurological diseases before or concomitant with the onset of a sABI (e.g., polytrauma with spinal cord lesion), a history of pre-existing FI or bowel inflammatory disease, lower bowel tract surgery, or pelvic cancer were excluded. The neurorehabilitation program included a scheduled and individualized diet, control of systemic and localized infections, and management of bladder disorders, bowel disorders, autonomic disorders, postural control, and mobility impairment. Patients underwent 3 h of rehabilitative treatment daily. In addition, speech/swallowing therapy was performed daily. Patients were treated daily with passive joint mobilization and placed upright on a tilt table. Moreover, sABIs were classified upon admission using the Italian version of the Rancho Level of Cognitive Functioning Scale (LCFS) [28,29].

The LCFS is one of the earliest developed scales for assessing cognitive functions after sABI. This scale classifies outcomes into eight levels, from no response (level I) to a purposeful and appropriate response (level VIII). The LCFS was administered on first admission and again at discharge.

The type of sABI was classified as traumatic/vascular/neoplastic/infection-related upon admission via a CT scan. Demographic variables and comorbidities such as frontal lobe lesions, pelvic fractures, paroxysmal sympathetic hyperactivity, *Clostridioides difficile* infections, healthcare-associated infections (HAIs), the presence and duration of tube feeding (nasogastric tube (NGT), percutaneous endoscopy gastrostomy (PEG)) upon admission and during recovery, length of hospitalization, and type of discharge were recorded. Fecal impaction was ascertained through a standard digital rectal examination consisting of three specific phases: (1) inspection of the anus and the neighboring tissues; (2) evaluation of the anocutaneous reflex; and (3) digital palpation. With the patient oriented in the left lateral position and the hips flexed at a 90° angle, an in-depth inspection of the anus and its surrounding tissues was undertaken. This inspection was designed to determine the presence of skin excoriation, skin tags, anal fissures, scars, or hemorrhoids and was conducted with appropriate lighting. Furthermore, an assessment of the sacral region was conducted: sacral reflexes required gently stroking the skin proximate to the anal area, progressing inward from all four quadrants, utilizing a stick tipped with a cotton swab. A standard reaction resulted in the swift contraction of the perianal skin, anoderm, and the external anal sphincter. This described reflex response is typically known as the anocutaneous reflex. If the cotton swab did not induce a reaction, the stick’s wooden end was used to invoke a more intense and distinct sensation. Following this, digital palpation was performed by methodically inserting a lubricated, gloved index finger into the rectal chamber. Assessing the resting sphincter tone consisted of categorizing its condition as normal, diminished, or augmented.

**Figure 1:**
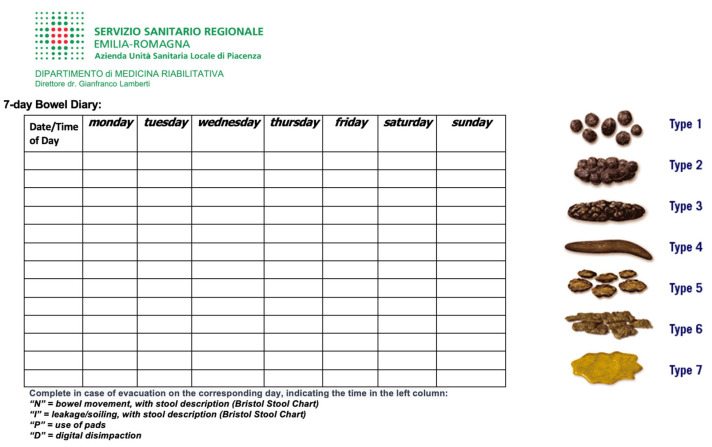
Bowel diary.

A three-week bowel diary assessed bowel behavior and was recorded upon admission and a week before discharge [30,31] (Figure 1).

This study aimed to determine whether new-onset FI episodes during the neurorehabilitation program undertaken after an acute sABI are a predictor for discharge settings and whether this condition and its persistence at discharge are dependent on clinical conditions during the rehabilitation phase.

Patient data were coded to identify certain factors—including feeding mode, sABI etiology, LCFS upon rehabilitation admission, FI upon rehabilitation admission, frontal lobe lesions, pelvic lesions, paroxysmal sympathetic hyperactivity, *C. difficile* infections, and healthcare-associated infections as independent variables—that could potentially influence the persistence of FI after an sABI at discharge from rehabilitation (the dependent variable), and whether FI is a predictor for the type of discharge setting.

Data were processed using JMP10 (SAS Institute Inc., Cary, NC, USA). Multivariable logistic regression was used for continuous variables, and chi-squared tests were used for nominal or categorical variables. Statistical analysis was completed in consultation with a biostatistician.

## 3. Results

A total of 531 patients (156 women and 365 men; median age, 48 years) consecutively admitted to our neurorehabilitation unit were included in this study. Ten patients were excluded because of pre-existing FI (*n* = 3), concomitant spinal cord injury (*n* = 3), previous colorectal surgery (*n* = 2), or irritable bowel syndrome (*n* = 2). The mean time from the severe brain injury to admission was 27.6 ± 11.8 days, with a mean length of stay in the neurorehabilitation unit of 70.5 ± 35.9 days. Table 1 shows the baseline demographic and clinical characteristics of the patients.

Upon admission, 443 (85%) patients showed FI, of whom 38% had a traumatic sABI. The number of FI episodes upon admission per week was 8 ± 6.3 (mean ± SD). The percentage of patients affected by FI who showed a partial recovery of consciousness level (LCFS ≥ 3) at admission to rehabilitation was 62.7%.

The patients were mainly affected by frontal lobe lesions (40.1%). Concomitant pelvic lesions in polytrauma were observed in 10.9% of the cases, and episodes of paroxysmal sympathetic hyperactivity were observed in 18.8% of the cases. Hospital-care-related infections were documented in 30.3% of patients with sABI, of whom 7.8% experienced *C. difficile* infections. During hospitalization, 26 deaths occurred, mainly from sepsis and neurological relapses.

Many patients (72.8%) were fed using PEG during recovery, whereas 18.2% were fed orally or with a mixed regimen. At discharge, 53.3% (264/495) of patients still had FI. Of those, 75.4% (199/264) had LCFS scores ≥ 3. The number of FI episodes at discharge in the last week was 4 ± 4.3 (mean ± SD).

When using dichotomous variables of any predictors that could modify the probability that the patient had FI at discharge (whether it was present at the time of admission or not), the logistic analysis only highlighted the presence of frontal lesions, the presence of autonomic crises in the acute phase, and LCFS scores at discharge as variables that were hierarchically more important, thus classifying others as less important (Table 2).

On the other hand, the following variables were not deemed significant: cause of sABI; LCFS; presence of FI and pelvic lesions (data collected at admission); duration of feeding via PEG or NGT in the acute phase; persistence of paroxysmal sympathetic hyperactivity; and presence of related infections at the time of treatment (data collected during the rehabilitation phase).

Patients with frontal lesions had a significantly higher risk of FI at discharge than those without lesions (*p* < 0.0001). The presence of autonomic crises in the acute phase further increased a patient’s chances of FI at discharge, with a doubled probability compared to patients who did not show paroxysmal sympathetic hyperactivity in the acute phase (*p* < 0.0001).

Regardless of the other predictors’ configurations, an increase in the LCFS level (increased level of cognitive function) from baseline was correlated with a reduction in the probability of FI at discharge. Research on potential correlations with various clinical factors using multiple regression to highlight predictors that can determine a change in the LCFS score at discharge, compared with those obtained at the time of admission, isolated the etiology, LCFS at admission, duration in days of feeding via NGT or PEG, and persistence of autonomic crises as hierarchically more relevant variables (Table 3).

Higher LCFS scores at admission were correlated with higher scores at discharge. Regarding etiology, the most significant increase correlated with traumatic pathogenesis, while anoxic pathogenesis correlated with lower LCFS scores at discharge compared to admission. An increase in the duration of feeding via NGT or PEG had a consistent and decreasing effect on the LCFS score at discharge. In particular, FI at admission did not appear to correlate with a low LCFS score at discharge; these results are consistent with what is reported in the literature [13].

**Figure 2:**
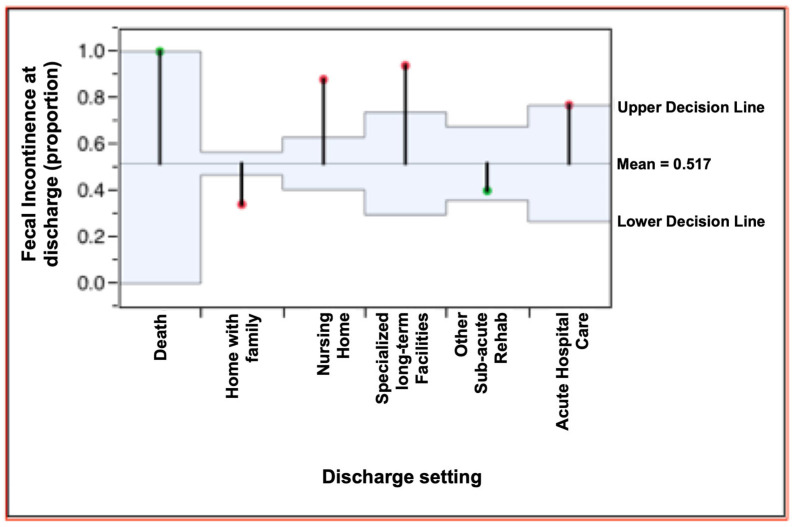
Fecal incontinence proportion and discharge setting: chi-squared analysis.

Finally, we evaluated whether the presence of FI at discharge influenced the post-rehabilitation destination of patients. The data in Table 3 indicate a difference between the proportion of patients with or without FI at discharge compared with the discharge setting, as shown in Figure 2. Among patients discharged to their homes at the end of the rehabilitation period, the proportion with persistent FI was lower than the general mean (34% vs. 53.3%); for those sent to long-term care, the proportion affected by persistent FI was higher than the general mean (87% vs. 53.3%). A similar trend was also found in transfers to NSV (vegetative state care), NAC (highly complex neurological care) (94% vs. 53.3%), and other acute care facilities (77% vs. 53.3%). The numbers of patients transferred to other rehabilitation facilities were similar to the general mean (40% vs. 53.3%). All of the findings above were statistically significant (*p* < 0.0001). Therefore, it is clear that there was a correlation between persistent FI at discharge and the setting to which patients were transferred (Figure 2).

## 4. Discussion

The present study was conducted in accordance with the STROBE statement [26]. The first interesting result was the high percentage of sABI patients with FI at admission to the neurorehabilitation unit, with an overall percentage of approximately 85.5% and approximately 62.7% for patients who recovered with an LCFS ≥3 (“localized response”). This partially diverges from the literature, where FI prevalence is estimated between 2.2% and 20.7% and reaches 50% in institutionalized individuals [31,32,33]; during the acute phase, FI has been reported to be between 23% and 70% [3,4,5] and persist during the chronic phase for 5% to 15% of patients [6,7,8,9,10,11,12,13,14,15]. A possible explanation is that our cohort also included participants with consciousness disorders, which may have negatively influenced our clinical evaluation.

Concerning the correlation we observed between FI persistence at discharge and EN duration, it is known that sABIs involve increased energy expenditure, increased weight, and muscle mass loss, along with the risk of impaired immune defenses. Several factors contribute to the onset of NBD in response to severe comorbidities during the acute phase: hypoperfusion (the need to infuse large quantities of fluid to support cardiovascular function, particularly because of intestinal edema, with reduced absorption, mobility, and lymphatic stagnation); sedation; and the use of vasoactive agents, which contribute to a slower transit speed with a reduction in absorption and constipation in 70% of cases [34]. Early EN is a crucial therapeutic strategy for maintaining intestinal mucosa and microbiome integrity [35], together with the immune responses that depend on it [36]. However, these benefits are not considered cost-effective, given possible risks and adverse events (e.g., diarrhea, delayed gastric emptying, aspiration pneumonia) [37,38]. Even after the acute phase, with the continuation of EN, alterations in intestinal transit, gastric secretion, and impaired intestinal absorption may be observed, especially when the characteristics of the ingredients are not modified [39]. Simultaneous modifications of the brain–gut axis [40], with a significant reduction in the diversity of microbiome components and the appearance of dysautonomia following the release of noradrenaline after sABI [41], results in need to reevaluate etiological aspects, which are often connected exclusively to the lesion sites of brain injuries and the therapies in place rather than the actual intestinal changes.

Concerning the persistence of FI at discharge, in our study, the data correlated exclusively with frontal damage, autonomic crises in the acute phase, and the LCFS score at discharge. The lack of correlation with other clinical indicators, both in the acute phase and during rehabilitation, including healthcare-related infections, such as *Clostridioides,* appears significant. In our study, the presence of healthcare-related infections did not seem to correlate with FI persistence at discharge from the neurorehabilitation program. The published literature shows correlations between pulmonary infections and acute gastrointestinal failure (AGF) [42], probably due to a compromised mucosal immune system being common to both [43]. Another interesting aspect of AGF seems to be its correlation with frontal damage, which correlates with the persistence of FI at discharge from neurorehabilitation, according to our data [44]. A similar observation concerning gastrointestinal tract disorders has been reported in a study of right frontal lobe lesions of the limbic system and ventromedial hypothalamus, which are characterized by increased serotonin and noradrenaline values in the same areas [45]. Our data revealed a correlation between the persistence of FI at discharge and the presence of paroxysmal sympathetic hyperactivity (PSH) in the acute phase. The hyperadrenergic syndrome is secondary to alterations of the central autonomic pathways rather than focal damage and is caused by a lack of hypothalamic inhibition of sympathetic and even non-nociceptive output, with a disconnection of the insular cortex [45]. PSH commonly presents with symptoms of tachycardia, excessive sweating, tachypnea, and increased temperature and is often associated with postural changes in decortication or decerebration [46]. After sABI, 60–90% of patients have at least one episode of dysautonomia in the first weeks after the acute event [47], although it also occurs frequently during rehabilitation [48,49]. The persistence of dysautonomia does not appear to correlate with the presence of other severe symptoms [48]; although it is frequently associated with disturbances of consciousness and extended hospital stays, its influence on prognosis is uncertain [48,50,51]. This is also consistent with the correlation we observed between the persistence of FI and the LCFS score at discharge. LCFS has been proposed and validated for monitoring functional and cognitive recovery after sABI [52]. Therefore, we believe that our results should not be interpreted as a direct correlation between PSH, a low LCFS score, and persistent FI at discharge but rather as the coexistence of symptoms in patients with a more compromised clinical condition.

These data have already been observed in the literature: in a 5-years retrospective study, Safaz [15] reported that the mean Mini-Mental State Examination (MMSE) scores and the median LCFS scores of patients with fecal or urinary incontinence were the same as those without incontinence.

Regarding our observed correlation between discharge planning and persistent FI at discharge from neurorehabilitation, it has been reported that FI may be responsible for reduced social reintegration [12] and poor clinical recovery [22], increasing the need for institutionalization [53,54].

In patients with sABI, fecal incontinence presents significant challenges. It imposes a heightened care burden due to the need for regular hygiene management and monitoring, and associated secondary risks further complicate home care. Additionally, the psychological impact on caregivers and family members is profound. Given these complexities, FI often necessitates institutionalized care at discharge, as opposed to home settings.

A final consideration about FI management is also due: transanal irrigation (TAI), administered either by healthcare professionals or by appropriately trained caregivers, represents a useful tool to manage such a disabling impairment. This intervention provides a means to regularly irrigate the bowel, thereby scheduling evacuations by thoroughly emptying the distal colonic segment. As a result, it aids in preventing episodes of fecal incontinence between irrigation sessions, offering a structured approach to bowel care and enhancing patient comfort and dignity [55].

There are devices designed for TAI intended for bedridden patients. These devices feature a closed irrigation and fecal collection system, ensuring that bed linens and pads remain uncontaminated. This allows for a simplified and hygienic procedure. Such streamlining could also facilitate caregivers in the home-based management of hygiene [7,55,56]. In recent years, strategies for managing neurogenic lower intestinal dysfunctions have been progressively enriched with useful tools, such as TAI systems performed at the patient’s bedside. Nevertheless, there are no specific studies about TAI in patients with sABI. Therefore, the authors advocate the development of protocols for the management of FI in the ABI population that can include TAI as a specific tool for reducing the time and cost of hygiene management.

One limitation of our study was the absence of follow-ups, which may have resulted in inaccurately recording persistent FI after discharge. It would be interesting to evaluate how intestinal function changes after a change in setting at discharge, both in patients who return home and those who move to specialized facilities. The second limitation was the inability to analyze the intestinal microbiome in order to evaluate the impact of dysbiosis on the brain–gut axis and fecal continence.

Moreover, we used LCFS to assess cognitive functioning because this study was designed before 2005, and the CRS-R (Coma Recovery Scale-Revised, at the moment, the best standardized neurobehavioral assessment measure designed for patients with consciousness disorders) was validated in the Italian language in 2007. Currently, we are exploring the relationship between fecal incontinence and DOC using the CRS-R.

Another potential limitation is that the data were sourced solely from a single academic tertiary care hospital, possibly not encompassing the entire spectrum of sABI cases; for this reason, future studies should also involve additional sABI reference centers in order to conduct multi-center investigations.

Future research should examine the pathophysiological mechanisms of NBD in patients with sABI, with the aim of better understanding the issue and identifying optimal care and management strategies. For this reason, if subsequent prospective studies demonstrate that return of bowel and/or bladder continence and cognition are useful in predicting long-term sABI outcome, potential applications may include early determination of rehabilitation services targeted at specific functional deficits, vocational rehabilitation planning, intervention development, resource allocation, cost projection, and family counseling.

## 5. Conclusions

In conclusion, we found significant persistent FI after sABI, even in patients recovering from unconsciousness. Predictive factors for the persistence of FI upon discharge from neurorehabilitation were the frontal lesion’s location, the presence of autonomic crises in the acute phase, and the LCFS score at discharge. FI occurring after sABI and persisting after rehabilitation treatment was more likely in patients who were referred to long-term care facilities and residential units. These data seem to agree with the findings of previous studies [3,8], but the correlation found in this study seems to be more predictive of disability following sABI; thus, it mainly represents a consequence of reduced functional recovery rather than an independent risk factor for unfavorable outcomes after sABI [10].

According to our data, the role of FI in the setting at discharge seems to be a significant and independent risk factor, and in our opinion, this depends on the caregiving needs that fecal incontinence management requires.

A structured simultaneous evaluation plan, both for an early rehabilitative approach and for a positive prediction of recovery, is needed for both NBD and neurogenic urinary dysfunction [54,56].

## Figures and Tables

**Table 1:** Demographic and clinical data of study participants (*n* = 521).

Age		Years	Median
Total (*n* = 521)		46.8 ± 29.3	48 (18–97)
Female (*n* = 156)		49.5 ± 18.8	
Male (*n* = 365)		45.6 ± 19.4	
**Etiology of sABI**		**Frequency**	**Percentage (%)**
Road traffic accident		232	44.5
Other traumatic (e.g., fall, sporting accident)		58	11.1
Subarachnoid hemorrhage		37	7.1
Hemorrhagic		114	21.9
Anoxic		53	10.2
Neoplastic		6	1.1
Infectious		21	4.1
**LCFS (rehabilitation admission)**		**Frequency**	**Percentage (%)**
I	No response	1	0.3
II	Generalized response	81	15.5
III	Localized response	220	42.3
IV	Confused—agitated response	82	15.7
V	Confused—inappropriate–non-agitated	80	15.4
VI	Confused—appropriate	43	8.1
VII	Automatic—appropriate	5	0.9
VIII	Purposeful—appropriate	9	1.8
**Feeding mode (rehabilitation admission)**		**Frequency**	**Percentage (%)**
	Oral	95	18.2
	Transgastric	379	72.8
	Nasogastric	47	9
**Fecal incontinence (rehabilitation admission)**		**Frequency**	**Percentage (%)**
Yes		443	85
No		78	15
**Frontal lobe lesions (rehabilitation admission)**		**Frequency**	**Percentage (%)**
Yes		209	40.1
No		312	59.9
**Pelvic ring lesions (rehabilitation admission)**		**Frequency**	**Percentage (%)**
Yes		57	10.9
No		464	89.1
**Paroxysmal sympathetic hyperactivity (acute phase)**		**Frequency**	**Percentage (%)**
Yes		98	18.8
No		423	81.2
**Clostridioides difficile** **infections (acute phase)**		**Frequency**	**Percentage (%)**
Yes		41	7.8
No		480	92.2
**Healthcare-associated infections (HAIs, acute phase)**		**Frequency**	**Percentage (%)**
Yes		158	30.3
No		363	69.7
**Fecal incontinence (rehabilitation discharge; *n* = 495)**		**Frequency**	**Percentage (%)**
Yes		264	53.3
No		231	46.7

**Table 2:** Logistic model variables for fecal incontinence at discharge from rehabilitation. OR—odds ratio; CI—confidence interval; χ^2^—chi-squared value.

Variables	OR (95% CI)	χ^2^
Feeding mode, NGT, or PEG (rehabilitation admission)	1.68 (0.47–6.05)	0.4187
Etiology of sABIs	2.08 (0.40–10.79)	0.3798
LCFS (rehabilitation admission)	0.60 (0.13–2.75)	0.5884
Fecal incontinence (rehabilitation admission)	1.13 (0.41–3.14)	0.8033
Frontal lobe lesions	33.83 (13.92–93.40)	*p* < 0.0001
Pelvic ring lesions	0.49 (0.14–1.60)	0.245
Paroxysmal sympathetic hyperactivity (acute phase)	2.08 (0.93–4.73	*p* < 0.0001
*Clostridioides difficile* infections (acute phase)	1.26 (0.29–5.52)	0.7608
Feeding mode, NGT, or PEG (duration)	0.11 (0.06–3.13)	0.1069
LCFS (rehabilitation discharge)	0.02 (0.01–0.07)	*p* < 0.0001
Paroxysmal sympathetic hyperactivity (persistence in the rehabilitation phase)	0.73 (0.22–2.45)	0.6147
Healthcare-associated infections (HAIs, rehabilitation phase)	(0.21–4.09)	0.9318

**Table 3:** Regression model variables for LCFS at discharge.

Variables	DF	Wald	F-Value	*p*
Feeding mode NGT or PEG (rehabilitation admission)	2	6.28	1.16	0.31
Etiology of sABIs	7	139.93	7.41	<0.0001
LCFS (rehabilitation admission)	1	463.04	171.67	<0.0001
Fecal incontinence (rehabilitation admission)	1	0.20	0.07	0.78
Frontal lobe lesions	1	0.11	0.04	0.83
Paroxysmal sympathetic hyperactivity (acute phase)	1	7.99	2.96	0.08
Pelvic ring lesions	1	0.41	0.15	0.69
Healthcare-associated infections (HAIs, acute phase)	1	3.08	1.14	0.28
*Clostridioides difficile* infections (acute phase)	1	1.54	0.57	0.45
Feeding mode (duration)	1	67.64	25.07	<0.0001
Paroxysmal sympathetic hyperactivity (persistence in the rehabilitation phase)	1	20.7	7.67	0.005
Healthcare-associated infections (HAIs, rehabilitation phase)	1	0.26	0.26	0.60
*Clostridioides difficile* infections (rehabilitation phase)	1	5.69	2.11	0.14

## Data Availability

All supporting data are presented in the manuscript.
